# An Inertial Sensor-Based Method for Estimating the Athlete's Relative Joint Center Positions and Center of Mass Kinematics in Alpine Ski Racing

**DOI:** 10.3389/fphys.2017.00850

**Published:** 2017-11-01

**Authors:** Benedikt Fasel, Jörg Spörri, Pascal Schütz, Silvio Lorenzetti, Kamiar Aminian

**Affiliations:** ^1^Laboratory of Movement Analysis and Measurement, Ecole Polytechnique Fédérale de Lausanne, Lausanne, Switzerland; ^2^Department of Sport Science and Kinesiology, University of Salzburg, Hallein-Rif, Austria; ^3^Department of Orthopedics, Balgrist University Hospital, University of Zurich, Zurich, Switzerland; ^4^Department of Health Sciences and Technology, ETH Zürich, Zürich, Switzerland

**Keywords:** inertial sensors, center of mass, alpine skiing, movement analysis, body model, posture estimation, validation

## Abstract

For the purpose of gaining a deeper understanding of the relationship between external training load and health in competitive alpine skiing, an accurate and precise estimation of the athlete's kinematics is an essential methodological prerequisite. This study proposes an inertial sensor-based method to estimate the athlete's relative joint center positions and center of mass (CoM) kinematics in alpine skiing. Eleven inertial sensors were fixed to the lower and upper limbs, trunk, and head. The relative positions of the ankle, knee, hip, shoulder, elbow, and wrist joint centers, as well as the athlete's CoM kinematics were validated against a marker-based optoelectronic motion capture system during indoor carpet skiing. For all joints centers analyzed, position accuracy (mean error) was below 110 mm and precision (error standard deviation) was below 30 mm. CoM position accuracy and precision were 25.7 and 6.7 mm, respectively. Both the accuracy and precision of the system to estimate the distance between the ankle of the outside leg and CoM (measure quantifying the skier's overall vertical motion) were found to be below 11 mm. Some poorer accuracy and precision values (below 77 mm) were observed for the athlete's fore-aft position (i.e., the projection of the outer ankle-CoM vector onto the line corresponding to the projection of ski's longitudinal axis on the snow surface). In addition, the system was found to be sensitive enough to distinguish between different types of turns (wide/narrow). Thus, the method proposed in this paper may also provide a useful, pervasive way to monitor and control adverse external loading patterns that occur during regular on-snow training. Moreover, as demonstrated earlier, such an approach might have a certain potential to quantify competition time, movement repetitions and/or the accelerations acting on the different segments of the human body. However, prior to getting feasible for applications in daily training, future studies should primarily focus on a simplification of the sensor setup, as well as a fusion with global navigation satellite systems (i.e., the estimation of the absolute joint and CoM positions).

## Introduction

For the purpose of gaining a deeper understanding of the relationship between training load and health in competitive sports, an accurate and precise estimation of the athlete's kinematics is an essential methodological prerequisite (Soligard et al., 2016). External load such as competition time, movement repetition counts, speed, acceleration, etc. (Soligard et al., 2016) could thus be quantified based on the estimated athlete's kinematics. In the context of alpine skiing, a major aim of coaching is to optimize the skier's posture and, thus, the relationship between his center of mass (CoM) and his left and right feet (Tjørhom et al., 2007; Kipp et al., 2008; Spörri et al., 2012b). In order to formalize this concept, a previous study focused on the parameter “vertical distance,” the distance between the left or right foot and the skier's CoM, and the parameter “fore-aft position,” the projection of the vector relying the CoM with the left or right foot onto the snow surface (Spörri et al., 2012b). Earlier studies in alpine skiing primarily used video-based stereophotogrammetric systems to determine an athlete's kinematics on a ski track (Supej et al., 2003; Federolf, 2012; Spörri et al., 2012a,b, 2016b; Hébert-Losier et al., 2014). Under such in-field conditions, photogrammetric errors of <1.5 cm were reported (Klous et al., 2010; Spörri et al., 2016c). However, despite major advantages regarding accuracy, corresponding measurement setups are complex, capture volumes are limited to a few turns only, and post-processing is time consuming.

Accelerated by these limitations and recent advances in wearable measurement technology, in the last few years, differential global navigation satellite systems (GNSS) have gained substantial attention as being a valuable alternative for estimating absolute CoM kinematics in-field (Brodie et al., 2008; Lachapelle et al., 2009; Waegli and Skaloud, 2009; Supej, 2010; Gilgien et al., 2013, 2014a,b, 2015a,b, 2016; Supej et al., 2013; Fasel et al., 2016a; Kröll et al., 2016). A major challenge of this alternative approach is that the GNSS antenna cannot be placed on the CoM directly and, therefore, the relative position of the GNSS antenna with respect to the CoM needs to be estimated. Parallel to these developments, CoM kinematics were also approximated based on a single inertial sensor for both human (e.g., Esser et al., 2009; Peyrot et al., 2009; Myklebust et al., 2015) and animal (e.g., Pfau et al., 2005; Warner et al., 2010) motion analysis. The hypothesis of these studies was that the chosen sensor location would match the CoM location. While this hypothesis may be true for gait, it may be violated in certain sports where upper and lower limb movement may alter the CoM position relative to the chosen sensor location. For example, for cross-country skiing, Myklebust et al. (2015) reported average RMS differences between the true CoM position and a sensor located at the sacrum on S1 of up to 32 ± 4 mm.

In alpine ski racing, one approach to resolve the issue of the CoM moving relative to the sensor location is the use of a simple pendulum model as suggested by Gilgien et al. (2015b) and Supej et al. (2013). However, while providing reasonable estimates of the athlete's overall CoM kinematics, such a model could not estimate the athlete's posture, which is key for the understanding of the relationship between specific loading patterns and health in competitive sports. Another option might be the fusion or combination of GNSS with body worn inertial sensor systems (Brodie et al., 2008; Fasel et al., 2016a). In recent years, several experimental field studies considered these systems to estimate athlete's relative joint center positions and CoM kinematics (Brodie et al., 2008; Supej, 2010; Fasel et al., 2016a). Currently, there exists no validated commercial product estimating the CoM kinematics based on inertial sensors. However, in the context of alpine skiing only the study Fasel et al. (2016a) critically validated such a fusion under in-field conditions, implying a certain need for additional scientific evidence and further improvements of the underlying body model. Specifically, they were using segment lengths obtained from the optical reference system, the upper trunk was divided in two segments not following literature recommendations (e.g., Dumas et al., 2007), and arm movement was not considered.

Thus, based on the aforementioned current stage of knowledge, the first objective of this study was to expand the body model suggested by Fasel et al. (2016a) for the estimation of the CoM to a more comprehensive and scalable model and including the upper limbs. The second objective was to validate the relative positions for the upper and lower limb joint centers and the athlete's CoM obtained from the inertial sensors against a video-based stereophotogrammetric reference system. The third objective was to evaluate the benefits of adding the upper limbs to the CoM estimation. The fourth objective was to assess the sensitivity of the wearable system to detect changes in the equipment used and turn types performed.

## Methods

### Measurement protocol

**Figure 1:**
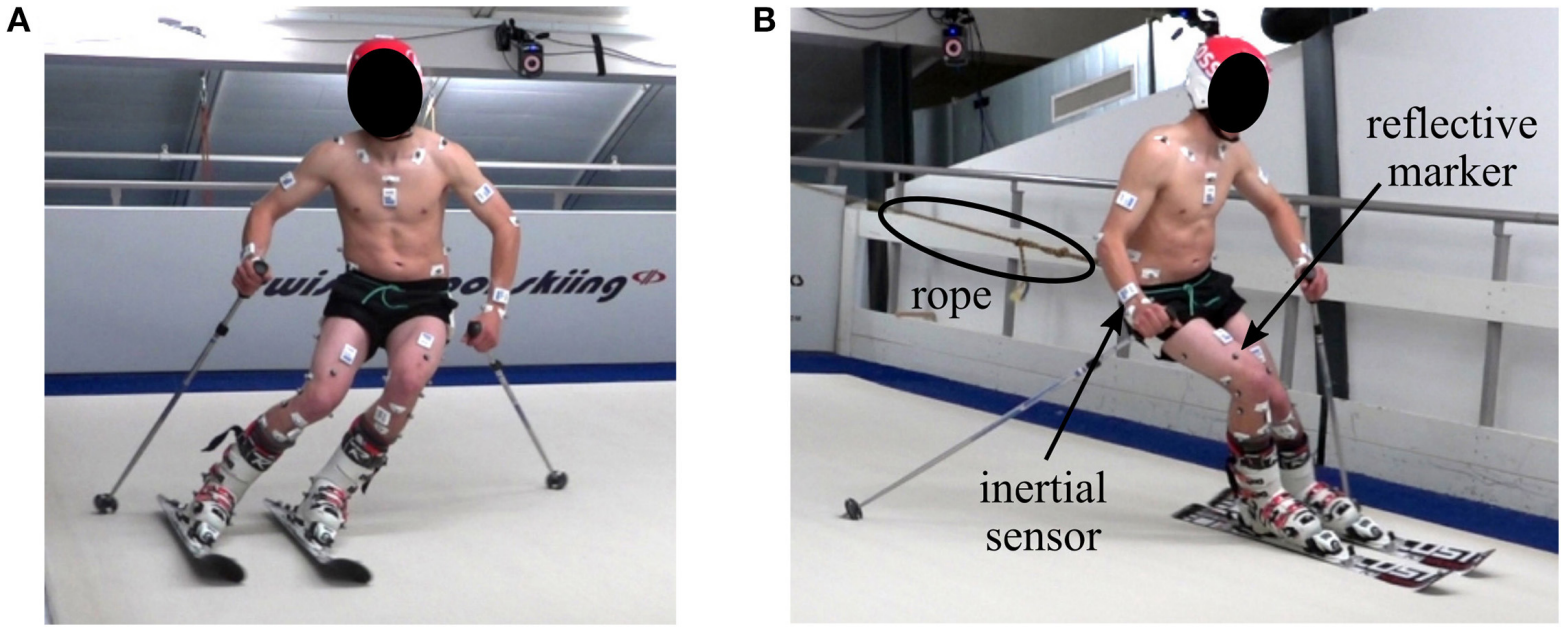
Illustration of the treadmill skiing setup. **(A)** Left turn, **(B)** right turn. To ensure that the athlete stayed in the capture volume, a rope connected a spring system with the athlete. The small white boxes are the inertial sensors and the gray dots the reflective markers.

The measurements were conducted on an indoor skiing carpet (Maxxtracks Indoor Skislopes, The Netherlands) with belt dimensions 6 × 11 m and 12° inclination (Figure 1). Eleven male competitive alpine skiers (20.9 ± 5.2 years, 176.1 ± 6.7 cm, 74.0 ± 10.9 kg) participated in the study. Written informed consent was obtained from all athletes prior to the measurements and the study was approved by the ethics committee of École Polytechnique Fédérale de Lausanne (Study Number: HREC 006-2016). Each athlete skied two trials with 140 cm long skis and two trials with 110 cm long skis at maximum belt speed of 21 km/h. Two types of skis were used to cover a broad range of different turn dynamics. Each trial lasted approximately 120 s and during the first half the athlete skied wide turns taking up the entire carpet width, while for the second half the athlete skied narrow turns taking up half the carpet width. Cones placed in the front of the treadmill were used to indicate the turn width. To ensure that the athletes stayed in the measurement volume, a spring system attached to a custom made belt pulled the athlete backwards (Figure 1).

### Reference system

**Figure 2:**
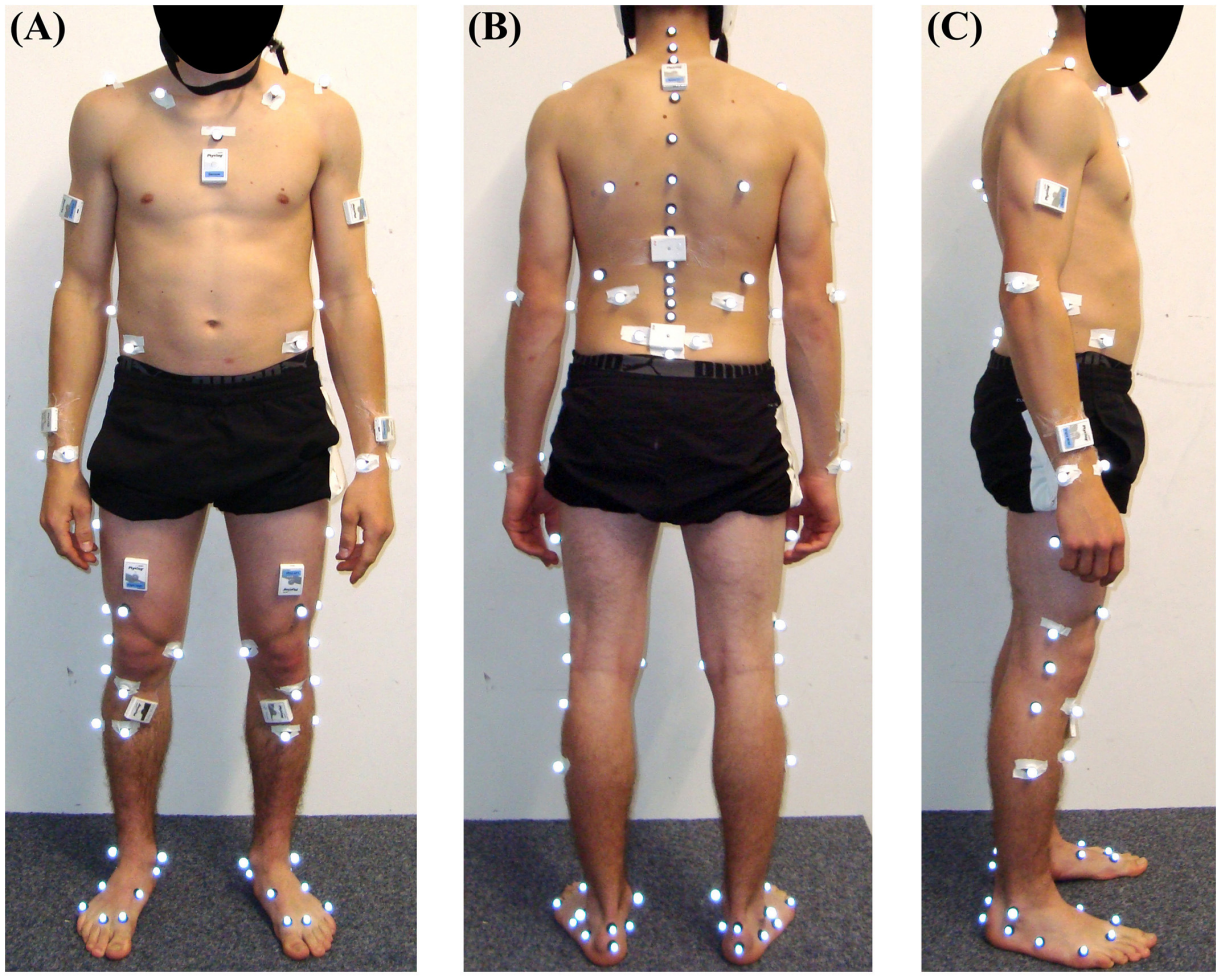
Sensor and marker setup from the front **(A)**, back **(B)** and side view **(C)**. The four markers fixed to the helmet are not shown here. The inertial sensors placed in the middle and upper back were not used for this study.

Ten infrared cameras (T160, Vicon Peak, UK) sampling at 100 Hz surrounded the carpet and covered the entire volume spanned by the carpet. The IfB marker set with 71 markers (List et al., 2013) (Figure 2) was used to obtain functionally determined ankle, knee, and hip joint centers and the 3D orientation of the shanks, thighs, pelvis, and lumbar, thoracic, and cervical trunk segments. Basic motion tasks as described in List et al. (2013) were performed to define the functional joint centers barefoot. The foot markers were then moved from the feet to the ski boots and a static posture was used to register the ski boot markers with the previously determined foot anatomical frame. Trunk markers were used to determine the trunk segments, as described in List et al. (2013). Since the IfB marker set could not directly measure upper limb joint centers, additional markers have been placed on the lateral humeral epicondyle, ulnar styloid, and radial styloid of both the left and right upper limbs. The shoulder joint center was defined to lie 3 cm below the acromion marker in the direction of the marker placed on the scapula inferior angle. The wrist joint center was defined to lie in the middle between the markers placed on the ulnar and radial styloids. The elbow joint center was defined to lie 3 cm to the medial direction with respect to the marker placed on the lateral humeral epicondyle. The medial direction has been defined to be normal to the plane spanned by the shoulder, wrist and lateral humeral epicondyle. In order to allow a comparison with the wearable model, the cervical joint center (CJC) and lumbar joint center (LJC) were estimated based on the anatomical tables from Dumas et al. (2007) scaled to the athlete height. CJC was estimated with respect to the marker placed on C7. LJC was estimated based on the average estimated LJC position with respect to the left and right hip joint centers. Four markers were placed on the athlete's helmet. Their mean position was used to approximate the position of the head vertex. Two markers were placed on each ski's tip and tail and allowed defining the skis' longitudinal axis. For the entire measurement in total 81 markers were attached to the participants. The segments' CoM were computed according to Dumas et al. (2007). The upper limb CoM was assumed to lie on the respective segment's longitudinal axes where the hand's longitudinal axis was the same as the forearm's longitudinal axis. The head's CoM was assumed to lie in the mid-point between the marker placed on C7 and the average position of the two markers fixed at the front of the helmet.

In order to allow a comparison to the inertial system, the joint and CoM positions were expressed relative to the LJC. The reference (global) coordinate system was defined as follows: the Y-axis was vertical, pointing upwards (e.g., vertical direction); Z-axis was horizontal and parallel to the treadmill-plane pointing to the right (e.g., lateral direction); the X-axis was the cross-product of the Z- and Y-axis and was pointing forwards (e.g., forwards slope direction in the horizontal plane).

The coaching-relevant parameters vertical distance and fore-aft position were computed according to Spörri et al. (2012b). For each leg (left and right) the vector *v*_*CoM, ankle*_(*t*) connecting the CoM with the ankle joint center was computed. The vertical distance was the norm of *v*_*CoM, ankle*_(*t*). The fore-aft position was obtained by the projection of *v*_*CoM, ankle*_(*t*) onto the line corresponding to the projection of ski's longitudinal axis on the snow surface. The snow surface was mathematically defined as the X-Z plane inclined by 12° around the Z-axis.

### Wearable system

Eleven inertial sensors (Physilog 4, GaitUp, Switzerland) were attached with adhesive tape to the shanks, thighs, sacrum, sternum, head, arms and wrists (Figure 2). Acceleration and angular velocity were measured at 500 Hz. Offset and sensitivity of the accelerometers were corrected according to Ferraris et al. (1995). To this end, each accelerometer was held static for a few seconds in the six positions where each sensing axis was either parallel, anti-parallel or orthogonal to the Earth's gravity field. Then a least-square fit was used to determine the sensors' offset and sensitivity such that the measured values would be 1, −1, 0, respectively. Offset of the gyroscopes was estimated during the standing still posture before each trial. The wearable system was synchronized with the reference system by an electronic trigger. The sensors' local frames were aligned with the segments' anatomical frames based on the functional calibration (squats, trunk rotation, hip abduction, and upright standing) described in Fasel et al. (2017b). In addition, the functional calibration of the arm sensors consisted of two movements, as illustrated on protocols.io (doi: 10.17504/protocols.io.jzncp5e): (1) slow arm movement in the sagittal plane where the hands hold a pole horizontally with both thumbs pointing medially. The hands were spaced approximately equal to the shoulder width and elbows were kept straight during the entire movement. Three movement cycles of up/down arm movement in the sagittal plane were performed. (2) Upright posture where the arms and wrists were kept vertically with straight elbows. The hands were oriented such that the palms were barely touching the thighs on their lateral side. For the functional calibration the following constraints were assumed: (i) the main rotation during the arm swing was supposed to occur along the medio-lateral axis of the arm and along the anterior-posterior axis of the wrist (e.g., forearm); (ii) the longitudinal axes of the arms and wrists were presumed to pass parallel to gravity during the upright posture.

#### Estimating segment orientation

Segment orientation was obtained based on the strap-down and joint drift correction as described in Fasel et al. (2017a,b). For initializing segment orientation, the athletes were standing straight, looking into the slope direction for 5 s before the treadmill was switched on. The wearable system's global frame was identical to the reference system's global frame and defined as follows: the Y-axis (e.g., vertical axis) was aligned with gravity, pointing upwards. X-axis (e.g., forwards axis) was perpendicular to gravity (i.e., horizontal) and pointing in the direction of the slope, facing downwards. The Z-axis (e.g., lateral axis) was the cross-product between the X- and Y-axis, pointing to the right. It was observed that, despite a standardized posture, the upper limbs' azimuths (i.e., direction of the segments' anterior-posterior axes) were not aligned. In order to find the segment's azimuths the same principle as for the joint drift correction presented in Fasel et al. (2017a,b) was used: after initial strap-down integration the segments' azimuths were assumed to be equal to the average joint acceleration orientation difference over the entire trial. Based on this principle, first the initial orientations of the arms were found with respect to the sternum. Second, the initial orientations of the wrists were found with respect to the arms. After this procedure orientation drift was corrected normally as in Fasel et al. (2017a,b). Example data and the matlab source code for the functional calibration, initial segment orientation estimation, and joint drift correction is available on Code Ocean (doi: 10.24433/CO.23792aee-07c5-4cdc-bfe9-9e85fa1bf5d5).

As no inertial sensors were placed on the skis, for computing the fore-aft position the ski orientations were estimated based on the shank orientations. To this end, it was assumed that the ankle was held in a constant position by the ski boot with a flexion of 17° without ankle abduction or internal rotation. In other words, the rotation between the ski's longitudinal axis and the shank's anterior-posterior axis was 17° around the shank's medio-lateral axis. The fore-aft parameters were then computed identically as for the reference system and described above.

#### Body model

**Figure 3:**
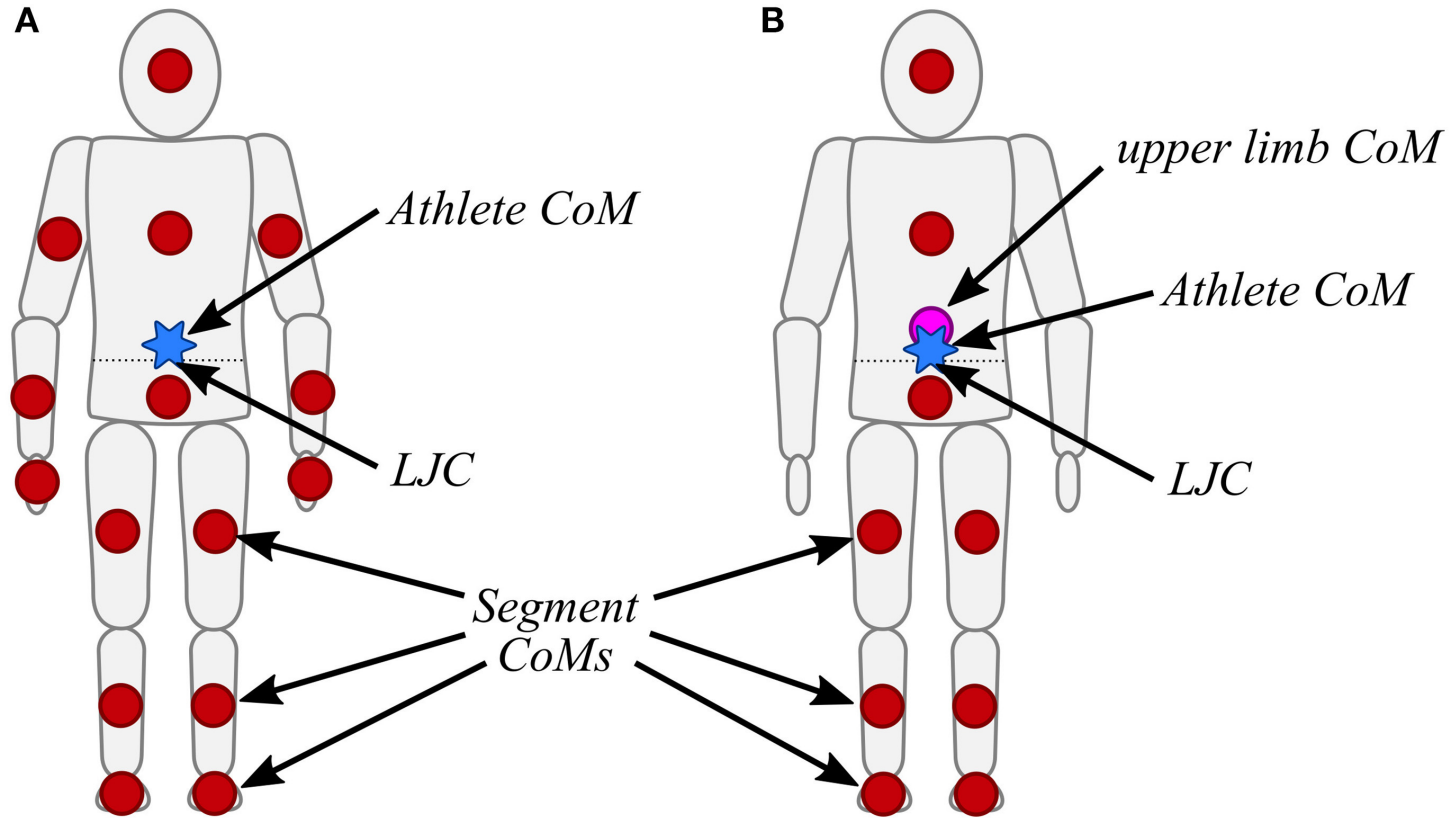
**(A)** Body model including the upper limbs. Each red circle represents a segment's CoM. The athlete's CoM is highlighted by the blue star. The LJC is indicated by an arrow and lies on the dotted line. **(B)** Simplified body model without the upper limbs. The approximated location of the upper limb's combined CoM is illustrated by the purple circle.

The body model was estimated based on a kinematic chain similarly to Fasel et al. (2016a). However, since the main aim of the body model was estimating the athlete's CoM, the origin of the kinematic chain was chosen as the LJC (Figure 3A). All segment dimensions were then defined according to Dumas et al. (2007), scaled for athlete height. It was assumed that the segment orientations obtained by the inertial sensors were identical to the anatomical frames of the corresponding segments. The trunk was modeled as two independent segments: pelvis and trunk. It was assumed that the pelvis orientation was equal to the sacrum orientation, and that the trunk orientation was equal to the sternum orientation. Thus, for example, the left hip joint position *p*_*left hip*_(*t*) was determined based on Equation (1) and the left knee position *p*_*left knee*_(*t*) based on Equation (2). All other joint positions were obtained with the same iterative way. Once the joint positions were known, the segment CoMs were estimated according to Dumas et al. (2007). In order to estimate the CoM of the hand, the hand was assumed to have the same orientation as the wrist. To estimate the foot CoM, it was assumed that the foot had the same orientation as the ski (i.e., 17° ankle flexion). A weight of 2 kg was added to each foot to take into account the weight of the ski boot. The skis were ignored for computing the CoM. The athlete's CoM was the weighted average of all segment CoMs. In a simplified model, without the arm and wrist sensors, the upper limbs' combined CoM was approximated at the relative position of (0.15, 0.10, 0.00 m) with respect to LJC expressed in the trunk's (i.e., sternum) anatomical frame (Figure 3B). The upper limb's relative CoM position was determined from average values of the full model and was scaled for athlete height with the same scaling factor as for the other segments.

(1) pleft hip(t)=Rsacrum(t) ∗ vleft hip

(2) $$p_{left\;knee}\left(t\right)=p_{left\;hip}\left(t\right)+{\;}_{\;}^{left\;thigh}R(t)\;*\;v_{left\;knee}$$

Where *t* is the time, Rsacrum(t) the orientation matrix of the sacrum, Rleft thigh(t) the orientation matrix of the left thigh, *v*_*left hip*_ the vector connecting the LJC to the left hip in the sacrum's anatomical frame, and *v*_*left knee*_ the vector connecting the left hip to the left knee in the left thigh's anatomical frame.

### Validation

A total of 44 trials (11 athletes, 4 trials per athlete) were analyzed. Error curves were computed by subtracting for each time sample the 3D position of the joint centers and CoM expressed relative to the LJC obtained with the reference system from the wearable system. For each trial, each individual axis and the total distance (i.e., the error norm), mean and standard deviation of the error were computed. Accuracy was defined as the group average of all trial mean errors and precision was defined as the group average of all trial standard deviations of the error.

The same error analysis was performed for the fore-aft parameters, whereas in addition Pearson's correlation coefficient was computed. For each trial 14 wide and 14 narrow turns were automatically segmented based on the crossing points of left and right vertical distance (i.e., norm of *v*_*CoM, ankle*_(*t*)) (Fasel et al., 2016b). For each turn the range of motion (RoM) of the vertical distance and the fore-aft position was computed and compared to the reference system with a Bland-Altman plot (Bland and Altman, 2007). Since the data points for the same trial were correlated, the limits of agreements (LoA) were computed as described in Fasel et al. (2017b). To assess whether the wearable system was sensitive to changes, Cohen's d was computed separately for the RoM obtained with the reference and the wearable system between trials (140 vs. 110 cm skis) and turn types (wide vs. narrow).

## Results

Errors for the left and right side were similar, thus, for the sake of clarity, in the following only the results for the left side are presented. Please refer to the appendix for the results of the right side.

Both accuracy and precision worsen for the more distal joint centers, and were worst for the ankles (total distance accuracy and precision of 109 and 30 mm) and wrists (total distance accuracy and precision of 97 and 16 mm) (Table 1). Standard deviation of the joint center accuracy was found to be between 6.3 and 57.6 mm. CoM accuracy and precision for the total distance were 25.7 and 6.7 mm, respectively.

**Table 1:** Average (standard deviation) accuracy and precision of the relative joint center positions along the X-axis (forwards slope direction), Y-axis (vertical direction), Z-axis (lateral direction), and total distance (norm of 3D difference).

**Joint center position**	**X-axis**		**Y-axis**		**Z-axis**		**Total distance**	
	**Accuracy**	**Precision**	**Accuracy**	**Precision**	**Accuracy**	**Precision**	**Accuracy**	**Precision**
Ankle	56.7 (57.6)	35.5 (14.5)	−16.3 (24.5)	20.8 (11.7)	23.1 (46.7)	48.4 (14.6)	109.1 (43.2)	29.7 (12.9)
Knee	26.2 (32.9)	25.3 (6.4)	21.8 (21.2)	20.8 (7.8)	40.0 (33.3)	34.6 (10.4)	79.7 (33.0)	18.9 (6.4)
Hip	−10.0 (10.1)	5.9 (1.6)	−3.8 (6.5)	4.7 (2.4)	21.4 (6.7)	5.1 (2.0)	28.1 (6.3)	4.7 (1.9)
CJC	−22.9 (28.1)	11.9 (3.4)	−5.9 (28.0)	9.1 (3.0)	−1.7 (35.9)	18.5 (5.1)	56.5 (24.7)	12.7 (4.7)
Head Vertex	−58.7 (39.2)	17.2 (6.1)	92.8 (56.6)	10.3 (3.4)	−3.3 (44.8)	25.5 (8.0)	127.3 (57.8)	16.9 (7.3)
Shoulder	−7.7 (31.9)	17.9 (4.8)	−69.0 (26.5)	14.0 (3.3)	−49.5 (28.8)	18.4 (5.7)	99.4 (24.3)	14.3 (4.6)
Elbow	14.0 (28.4)	17.3 (4.9)	−6.1 (30.5)	15.3 (4.6)	−9.4 (27.4)	17.5 (5.3)	55.1 (20.3)	14.7 (3.4)
Wrist	−50.8 (39.3)	20.4 (6.9)	−49.7 (35.7)	21.4 (7.2)	−14.4 (32.37)	21.4 (7.2)	97.0 (29.4)	16.3 (4.8)

*All units are mm*.

**Figure 4:**
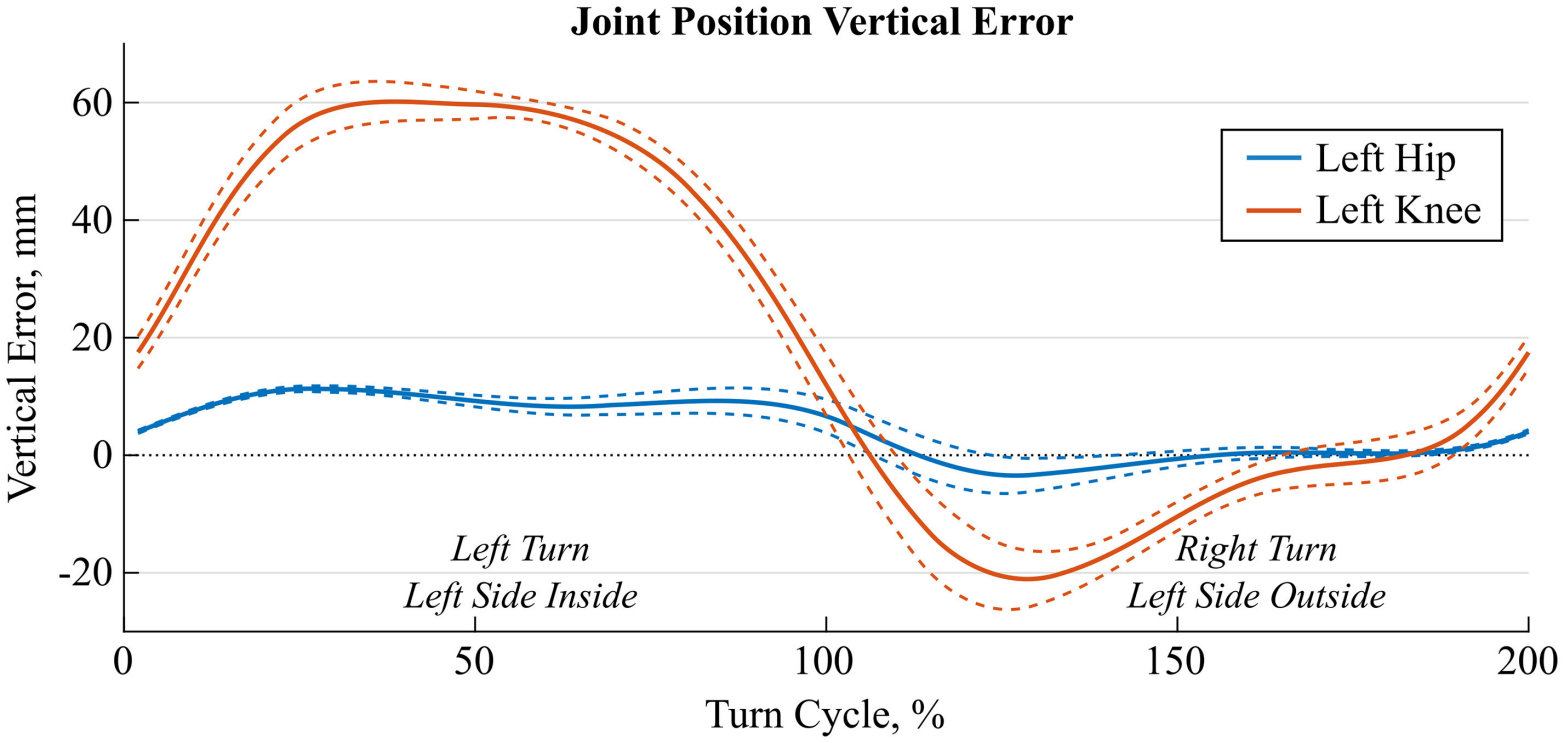
Average (solid lines) ± 1 standard deviation (dashed lines) of time-normalized hip (blue) and knee (orange) joint position errors along the vertical Y-axis for 9 left and right turns of a representative trial. The first 100% of the turn cycle is a left turn where the left leg is the inside leg and the second 100% is a right turn where the left leg is the outside leg.

Especially the knee and ankle joint position errors were dependent on the turn phase, i.e., were different for the inside than the outside leg. Figure 4 shows time-normalized errors for the knee and ankle joints for a typical athlete and nine wide left/right turns of the trial with 140 cm skis. While the hip's vertical position error (Y-axis) remained below 10 mm throughout the turn cycle, the knee joint position had large errors during left turns (i.e., for inside leg).

Accuracy and precision for the CoM computed with the full model was found to be <8.6 mm and <11.2 mm for each axis. Simplifying the model did not impact the CoM precision, but added a bias in the forwards and vertical direction, in which the CoM was estimated 8.5 mm too low and 13.5 mm too posterior (Table 2).

**Table 2:** Average (standard deviation) accuracy and precision of the relative CoM positions for the full model with arms and the simplified model without arms.

**CoM position**	**X-axis**		**Y-axis**		**Z-axis**		**Total distance**	
	**Accuracy**	**Precision**	**Accuracy**	**Precision**	**Accuracy**	**Precision**	**Accuracy**	**Precision**
Body model with arms	−8.6 (13.8)	6.4 (1.7)	0.6 (14.2)	4.5 (1.7)	−0.5 (13.1)	11.2 (3.3)	25.7 (10.9)	6.7 (2.2)
Body model without arms	−13.5 (12.2)	6.6 (1.6)	−8.5 (14.4)	4.5 (1.7)	−0.1 (12.5)	11.5 (3.5)	28.6 (9.6)	7.2 (2.6)

*All units are mm*.

**Figure 5:**
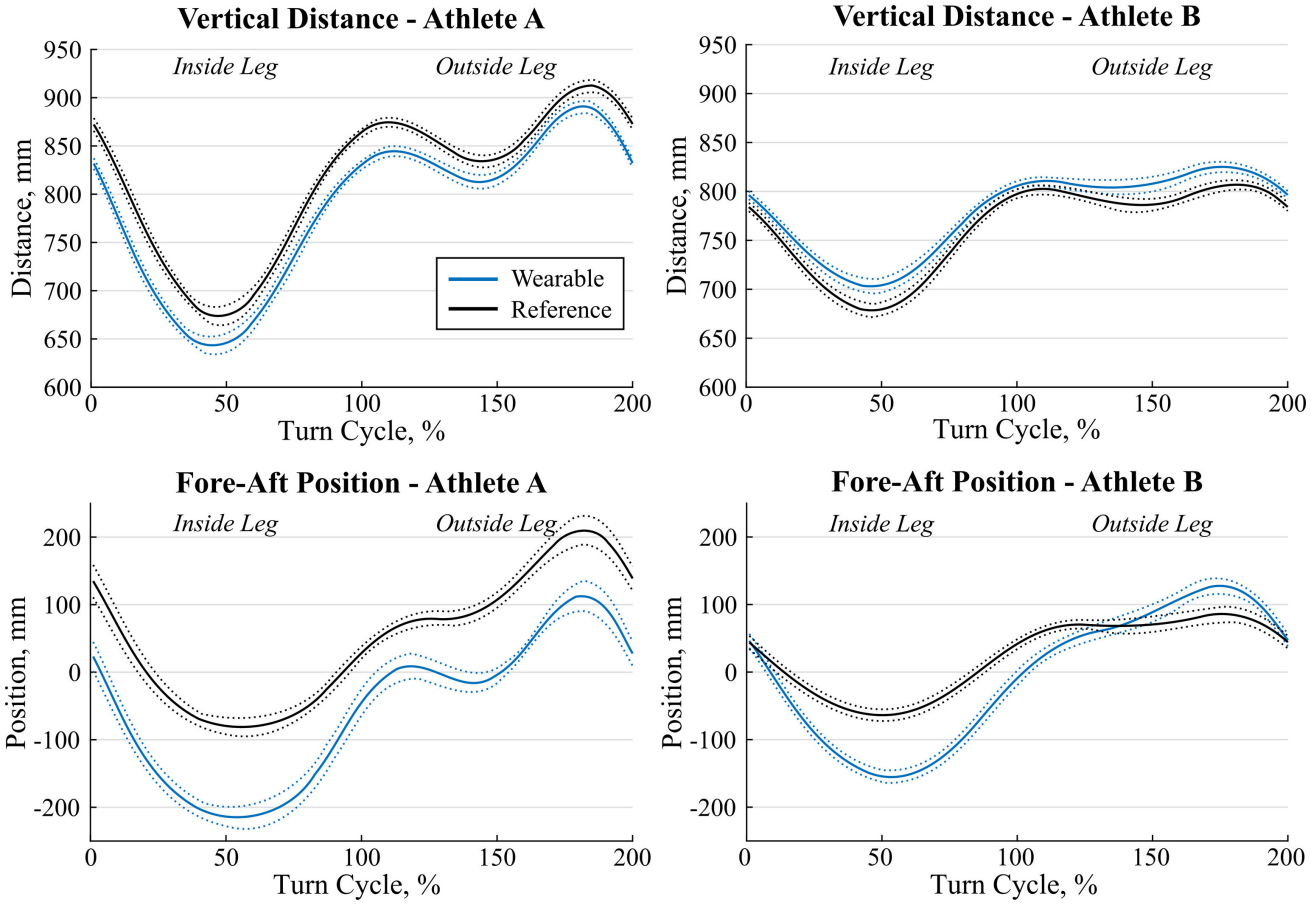
Average (solid lines) ± 1 standard deviation (dotted lines) of vertical distance **(top)** and fore-aft position **(bottom)** of the left leg for the same condition for two athletes A and B (left vs. right) and 14 wide double turns. The wearable system is shown in blue and the reference system in black. The first 100% of the turn were a left turn, thus the left leg was the inside leg. The second 100% of the turn were a right turn, thus the left leg was the outside leg.

For both the full and simplified models, correlation was >0.98 for the vertical distance and approximately 0.90 for fore-aft position (Table 3). For the full model, fore-aft position was underestimated by 74 mm on average and its average precision was 34 mm. For the full model, vertical distance was on average overestimated by 3 mm with a precision of 11 mm (Table 3). Errors were only slightly different for the simplified model. Figure 5 shows the average ± standard deviation curves for 14 wide double turns of two representative athletes. The full model was used to obtain the wearable curves.

**Table 3:** Average (standard deviation) accuracy and precision of the fore-aft parameters and their correlation to the reference system for the full model with arms and the simplified model without arms.

**Parameter**	**Body Model**	**Accuracy**	**Precision**	**Correlation**
Vertical distance	With arms	3.3 (19.8)	10.6 (5.4)	0.990 (0.010)
	Without arms	−5.5 (19.7)	10.9 (5.7)	0.989 (0.010)
Fore-aft position	With arms	−73.9 (47.0)	34.0 (11.0)	0.896 (0.087)
	Without arms	−76.7 (49.1)	33.8 (10.9)	0.897 (0.087)

*Units for accuracy and precision are mm*.

**Figure 6:**
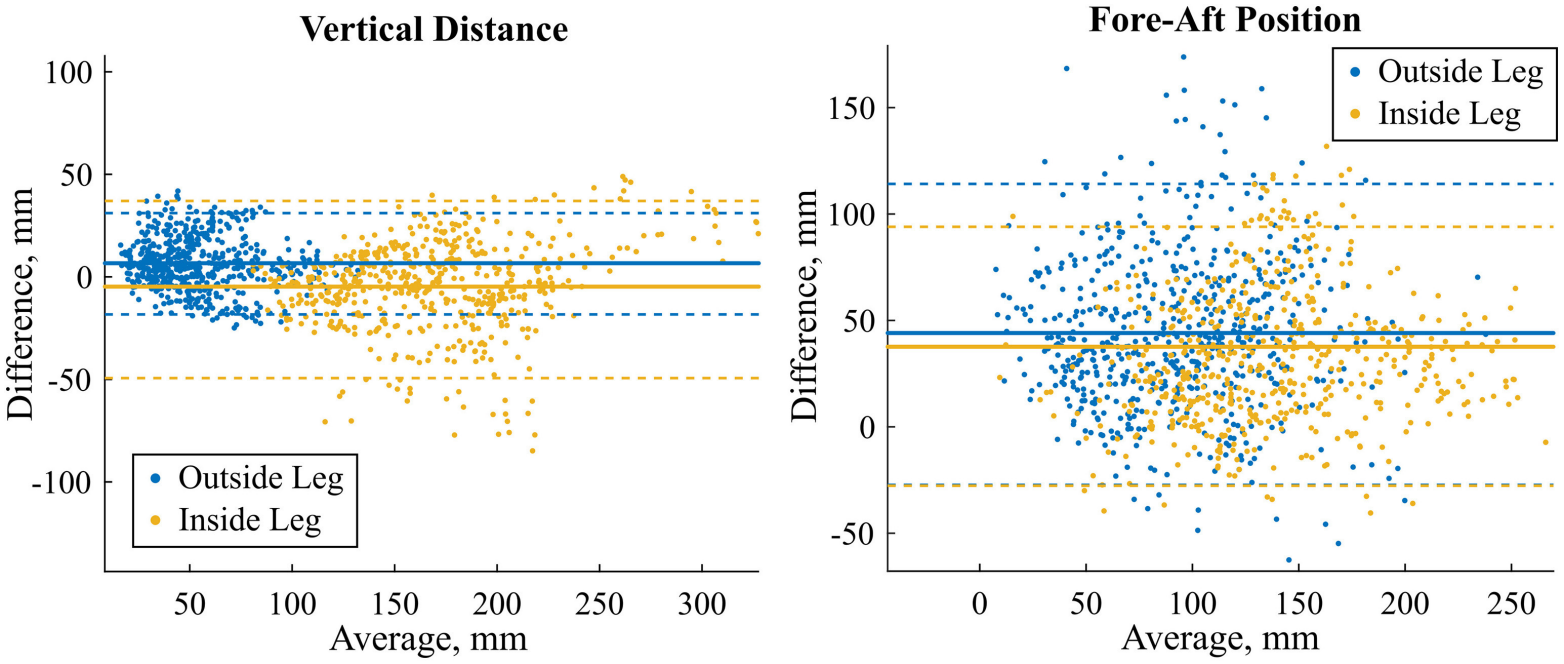
Bland-Altman plots for the range of motion of the vertical distance **(left)** and fore-aft position **(right)**. The model without arms was used to generate the figures and compute the LoA (dashed lines). Mean error is shown with the solid lines. Blue marks the outside leg and yellow the inside leg. LoA for both models and outside and inside legs are reported in Table 4.

LoA for the RoM of the vertical distance and fore-aft position were considerately lower for the outside leg than the inside leg (Table 4, Figure 6). The reference average value (standard deviation) of the vertical distance RoM was 53.8 mm (23.5 mm) for the outside leg and 168.9 mm (45.0 mm) for the inside leg. The reference average value (standard deviation) of the fore-aft position RoM was 92.7 mm (40.1 mm) for the outside leg and 136.7 mm (47.2 mm) for the inside leg. Cohen's d for the RoM computed with the reference system and the full model were similar: between wide and narrow turns >1 for the fore-aft position and >2 for the vertical distance. Simplifying the model by removing the arms did only slightly change the fore-aft parameters' accuracy and precision. As for the full model, Cohen's d were similar to the reference system.

**Table 4:** Limits of agreements (LoA) for the range of motion (RoM) of the vertical distance and fore-aft positions.

**Parameter**	**Body model**	**Errors outside leg**			**Errors inside leg**		
		**Lower LOA**	**Mean**	**Upper LoA**	**Lower LoA**	**Mean**	**Upper LoA**
RoM Vertical distance	With arms	−18.6	8.4	32.4	−49.1	−5.2	40.1
	Without arms	−17.8	8.4	30.9	−50.0	−5.8	37.8
RoM Fore-aft position	With arms	−26.8	48.9	117.6	−30.5	29.0	91.9
	Without arms	−29.4	47.9	117.3	−25.5	37.0	92.5

*All units are in mm*.

## Discussion

In the current paper, an inertial sensor-based method to estimate the athlete's relative joint center positions and center of mass (CoM) kinematics during alpine skiing has been proposed. In addition to these estimates, the joint center- and CoM-related measures “vertical distance” and “fore-aft position” were computed. The new method's validity was assessed by comparing it to an optoelectronic stereophotogrammetric reference system (gold standard). Accuracy (precision) for the CoM, vertical distance and fore-aft position were 25.7 mm (6.7 mm), 3.3 mm (10.6 mm), and −73.9 mm (34.0 mm), respectively. Excluding the upper limbs from the body model decreased the accuracy and precision of all curves by less than 3 mm, except for the vertical distance where the accuracy changed from 3.3 to −5.5 mm. The proposed procedure for estimating relative segment azimuth during posture initialization seemed sufficiently accurate and precise. Interestingly, the elbow joint position was estimated with better accuracy than the shoulder and wrist joint positions. However, prior to analyzing specific movements for which arm motion is key, the proposed orientation initialization should be validated more specifically.

### Joint center positions

As expected, errors of the relative joint positions increased along the kinematic chain. Two factors might have contributed to these errors: incorrect segment dimensions and inaccurate segment orientation estimations. Segment dimensions were taken from Dumas et al. (2007) and were scaled for athlete height only. Therefore, athlete-individual deviations from the model were not considered and led to a potential bias in the estimation of the segment length. As an example, our athletes had on average a 40 mm wider pelvis and 69 mm shorter trunk. Subject-specific anthropometric measurements could reduce this error; however, at the costs of a more complicated measurement procedure. Furthermore, segment orientation estimation errors might have directly affected joint estimation errors. For example, knee joint position errors were by a factor of 3–4 higher than for the hip joint. The large precision decrease observed could be attributed to soft tissue artifacts of the thigh. Actually, high muscle activation levels during the turns could have temporarily changed the sensor's alignment with respect to the underlying bone. In this context, it is known that during a turn the inside leg has higher hip and knee flexion angles but has to support less force (Klous et al., 2012; Kröll et al., 2015). Thus, it is reasonable that the muscle activation at the inside leg is different compared to the outside leg (Kröll et al., 2011), what, while turning, might have led to a different amount of soft tissue artifact and, therefore, different errors in the estimation of the thigh segment orientation (Figure 4). To overcome these limitations, soft tissue artifacts could be modeled for example with a double static calibration as proposed by Cappello et al. (1997), as well as by measuring different static postures with and without muscle pre-activation (e.g., upright standing or sitting on a chair).

### CoM position

Despite the limited performance of joint position estimation, CoM position was estimated with very good accuracy and precision. One explanation could be that errors from individual joint positions were averaged out when computing the athlete's CoM. Surprisingly, and in contrast to the findings from Eames et al. (1999) and Whittle (1997) for walking, removing the upper limbs from the model did not decrease CoM accuracy and precision significantly. One potential explanation for this observation might be the fact that during alpine skiing arm movements are mostly symmetrical and that (at least for the current indoor carpet skiing setup) the arms were almost held in a constant position. Another explanation might be the fact that the upper limbs contribute on average only 10% to total body mass (Dumas et al., 2007). Thus, even if arm movements may not have been estimated correctly, corresponding effects on CoM position are rather marginal.

### Vertical distance and fore-aft position

**Figure 7:**
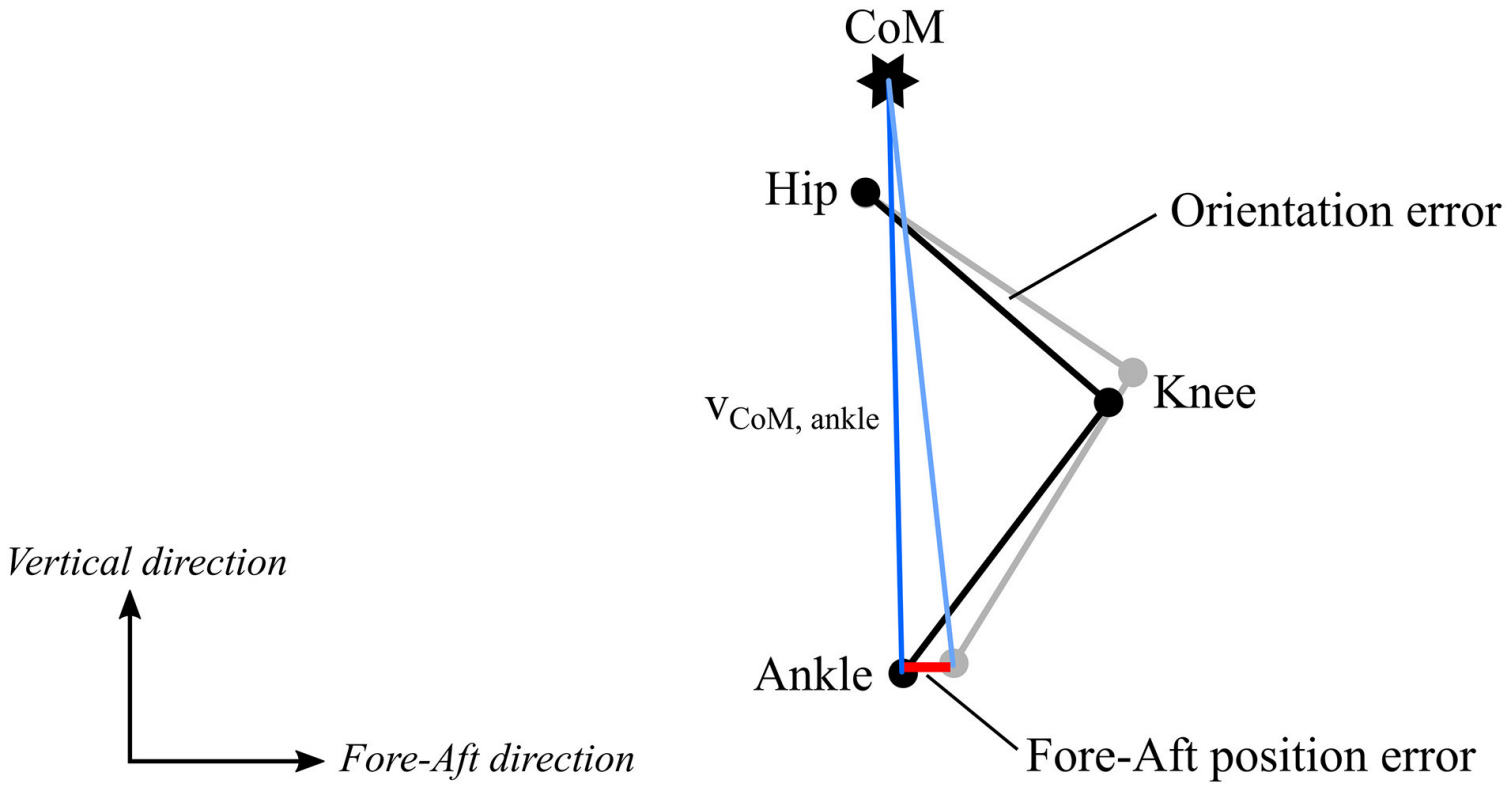
Influence of thigh orientation estimation error on ankle position. Black shows the original leg position and gray the leg position with a thigh and shank orientation error. The blue lines show the ankle – CoM vectors. The fore-aft position (projection of ankle – CoM vector onto the fore-aft axis) is more affected by this orientation error (difference shown in red) than the vertical distance (length of ankle – CoM vector).

Both vertical distance and fore-aft position were estimated with higher precision than reported previously in Fasel et al. (2015), underlining the better suitability of the revised body model used in the current study. Particularly, for the measure “vertical distance,” accuracy was slightly improved, while for the fore-aft position accuracy was slightly reduced. Moreover, compared to vertical distance fore-aft position was found to be more sensitive to ankle position errors (Figure 7). Under the hypothesis that the largest error source could be attributed to incorrectly estimated thigh orientation due to soft tissue artifacts, a change in thigh orientation would essentially affect the direction of the vector relying the ankle to the CoM, but not its length. Accordingly, soft tissue artifacts may only marginally alter the vertical distance, however, may substantially influence fore-aft position (Figure 7), why in the context of inertial-based measurements this parameter should be used with caution. However, future improvements regarding a reduction of the soft tissue artifacts might help to overcome these fore-aft position-related limitations. In this study, the snow surface was defined mathematically for both the reference and wearable system. For on-snow measurements this surface has to be estimated first, for example by constructing a 3D terrain model with drones (e.g., Pix4Dmapper, Pix4D, Switzerland).

### Methodological limitations

Despite the carefully chosen reference system and setup, the study has some limitations that are worth to be discussed: first, the model was specifically designed for lower limb and trunk motion capture. Accordingly, upper limb joints (shoulders, elbows, wrists) and head vertex were only approximately tracked. Especially for the shoulder joint and head vertex reference positions might have been estimated with errors of up to a few centimeters. This inaccuracy was judged to be acceptable, since a validation of the upper limb position and orientation was not the main aim of this study. Second, for the estimation of CoM, segment inertial parameters were taken from Dumas et al. (2007) and were only scaled to athlete height. However, the body model could be further individualized by taking into account the athlete's segment lengths and an estimation of their muscle masses. Third, as inertial sensors cannot provide absolute position measurements, only the relative joint and CoM positions were validated. For reasons of convenience, the lumbar joint center (LJC) has been defined as the origin for both systems, even though it could not be measured directly by the reference system. However, by averaging the LJC estimated from the left and right hip joint center, measurement errors were aimed to be minimized. Fourth, the ecological validity of the study might be limited. Despite the fact that the movement patterns on the treadmill are known to correspond well to the real on-snow skiing situation (Spörri et al., 2016a), the reduced speed might have led to less dynamic movements and less arm motion. Moreover, vibration from skidding on the snow did not exist either. Therefore, it is expected that errors for on-snow skiing might be slightly larger than presented here.

### Perspective

Overall, based on the system's accuracy and precision and, specifically, based on Cohen's d, the proposed method was found to be sensitive enough to distinguish between different types of turns (wide/narrow). Thus, the current method may also provide a useful information for monitoring and controlling adverse external loading patterns that occur during regular on-snow training. Moreover, as demonstrated earlier and in other settings (Chardonnens et al., 2012, 2014; Rawashdeh et al., 2016; Yu et al., 2016; Whiteside et al., 2017), such an approach is also suitable for quantifying competition time, movement repetitions and/or the accelerations acting on the different segments of the human body. However, prior to getting feasible for applications in settings of daily training, future studies should primarily focus on a simplification of the sensor setup, as well as a fusion with global navigation satellite systems (i.e., the estimation of the absolute joint and CoM positions). It has to be pointed out that, in order to fully quantify the total load, not only the external but also the internal load should be quantified (Soligard et al., 2016).

## Conclusion

The system allowed computing the athlete's relative joint center and CoM position with sufficient accuracy and precision for detecting meaningful difference in alpine skiing. Only the accuracy and precision of the most distal joints (e.g., ankle) are on the limit of an acceptable range. The accuracy and precision of the ankle positions can be considered acceptable for computing the vertical distance, but not for calculating the fore-aft position. Future developments should aim at reducing soft tissue artifacts such that knee and ankle positions could be estimated with better precision. To compute the absolute CoM position with respect to a fixed global reference frame, the obtained relative CoM position and body model could be combined with an absolute position of a body part (e.g., head), for example measured with differential GNSS. A future study should also address how to simplify the system so that it could be used for everyday external load monitoring, with fully automated calibration and data analysis.

## Author contributions

BF, JS, PS, SL, and KA conceptualized the study design. BF, JS, PS, SL conducted the data collection. BF, JS, PS contributed to the analysis and interpretation of the data. BF drafted the manuscript, all other authors revised it critically. All authors approved the final version and agreed to be accountable for all aspects of this work.

### Conflict of interest statement

The authors declare that the research was conducted in the absence of any commercial or financial relationships that could be construed as a potential conflict of interest. The reviewer TS declared a shared affiliation, with no collaboration, with one of the authors JS to the handling Editor.
